# Complementary relation of quantum coherence and quantum correlations in multiple measurements

**DOI:** 10.1038/s41598-018-36553-3

**Published:** 2019-01-22

**Authors:** Zeyang Fan, Yi Peng, Yu-Ran Zhang, Shang Liu, Liang-Zhu Mu, Heng Fan

**Affiliations:** 1 School of Astronautics, Harbin Institute of Technology, Harbin, 150001 China; 2 Beijing National Laboratory for Condensed Matter Physics, Institute of Physics, Chinese Academy of Sciences, Beijing, 100190 China; 3 School of Physical Sciences, University of Chinese Academy of Sciences, Beijing, 100190 China; 4 Beijing Computational Science Research Center, Beijing, 100094 China; 5 Theoretical Quantum Physics Laboratory, RIKEN Cluster for Pioneering Research, Wako-shi, Saitama, 351-0198 Japan; 6 School of Physics, Peking University, Beijing, 100871 China; 7 CAS Center for Excellence in Topological Quantum Computation, University of Chinese Academy of Sciences, Beijing, 100190 China

## Abstract

Quantum coherence and quantum correlations lie in the center of quantum information science, since they both are considered as fundamental reasons for significant features of quantum mechanics different from classical mechanics. We present a group of complementary relations for quantum coherence and quantum correlations; specifically, we focus on thermal discord and conditional information in scenarios of multiple measurements. We show that the summation of quantum coherence quantified in different bases has a lower bound, resulting from entropic uncertainty relations with multiple measurements. Similar results are also obtained for thermal discord and for post-measurement conditional information with multiple measurements in a multipartite system. These results indicate the general applications of the uncertainty principle to various concepts of quantum information.

## Introduction

Quantum coherence, namely a principle of superposition of quantum states, is one of the cornerstones of the quantum theory. It is believed to provide advantages in tasks of quantum information processing and quantum computation over classical methods. Recently, the rigorous framework of the quantification of quantum coherence has been introduced with several criteria proposed for any coherence measure to satisfy^1–17^. Based on this framework, different quantitative studies of quantum coherence have also attracted many attentions^18–26^, see reviews^27–29^ for progresses and references. Furthermore, quantum coherence is closely related to various quantum correlations, such as entanglement and quantum discord^30–33^. In this paper, we focus on two well known quantum correlations: thermal discord^34–36^ and conditional information^37^. Also known as one-way deficit, thermal discord is often studied in the quantum thermodynamics, e.g., the research of Maxwell’s Demon^35,36^. Conditional information plays an important role in quantum entanglement^38–40^ and state merging^41,42^, because a negative value of conditional information signals the existence of entanglement. Apart from these quantum quantities, the Heisenberg uncertainty principle^43–51^ has been widely studied in the quantum information processing. These studies, including entropic complementary relations^46–51^, not only provide a deeper understanding of quantum mechanics, but also give a useful tools for researches on the quantum information processing.

In sharp contrast to quantum entanglement that is invariant for any local unitary transformation, the quantification of quantum coherence is in general basis dependent and depends on both the quantum state itself and the basis we choose. It is then an interesting question whether we can find a class of basis-independent coherence measures or diminish the effects of the basis chosen. Based on this consideration, in this work, we investigate the total quantum coherence for multiple bases and present the results in forms of complementary relations. We start from uncertainty relations^49,51^ with measurements on different subsystems and multiple measurements. We obtain a group of complementary relations for quantum coherence, thermal discord and conditional information, which give a deeper understanding of these concepts. Explicitly, complementary constraints are given on quantum coherence and basis-dependent thermal discord with respect to multiple measurement bases. As an example, we investigate quantum coherence in a one-particle system and thermal discord in a bipartite system by implementing all the measurements on a single subsystem. In addition, for multiple measurements in a multipartite system, we also present a complementary relation for post-measurement conditional information.

## Results

### Coherence quantification and the definitions of thermal discord and conditional information

A natural measure of quantum coherence is defined as a pseudo-distance formulated by the relative entropy between the studied quantum state with the nearest incoherent state. It can be proven that this nearest incoherent state is the corresponding diagonal matrix of the studied density matrix with all off-diagonal elements zero^1^,

1 $${C}_{{\rm{RE}}}^{( {\mathcal M} )}(\rho )=S({\tilde{\rho }}^{( {\mathcal M} )})-S(\rho ),$$

where *S*(*ρ*) := −Tr*ρ* log_2_*ρ* is the von Neumann entropy, ${\mathcal M} \,:=\{{\Pi }_{i}\,:=|i\rangle \,\langle i|\}$ is the projective measurement, and ${\tilde{\rho }}^{( {\mathcal M} )}\,:={\sum }_{i}{{\rm{\Pi }}}_{i}\rho {{\rm{\Pi }}}_{i}$ is the post-measurement state. It is apparent that the post-measurement state ${\tilde{\rho }}^{( {\mathcal M} )}$ is the diagonal matrix of the density matrix *ρ*, ${\tilde{\rho }}^{( {\mathcal M} )}={\rho }_{{\rm{diag}}{\rm{.}}}$. In this sense, the relative entropy of coherence in Eq. (1) can be understood as the increase of the entropy via a projective measurement ${\mathcal M}$, meaning the difference of the entropy between the post-measurement state and the original state. Therefore, quantum coherence describes the quantum resource destroyed in the projective measurement, which is naturally interpreted from the projective-measurement point of view. Because quantum coherence measures such as ${C}_{{\rm{RE}}}^{( {\mathcal M} )}(\,\cdot \,)$ are basis dependent, finding the relation between quantum coherence of the same state with respect to different measurement bases ${\mathcal M}$ would be significant. By varying the measurement basis, quantum coherence changes and has a maximum^52^. Then, considering multiple measurements, it is not clear what is the relationship among those quantifications of quantum coherence based on different bases. We will derive those relations using entropic uncertainty inequalities for multiple measurements with and without the quantum memory.

Let us consider a bipartite state *ρ*_A*B*_ with two subsystems A and B. The projective measurement ${ {\mathcal M} }_{{\rm{A}}}^{(k)}\,:=\{{{\rm{\Pi }}}_{{\rm{A}}i}^{(k)}\,:=|{i}^{(k)}{\rangle }_{{\rm{A}}}\langle {i}^{(k)}|\}$ is performed on the subsystem A, and we have the unnormalized post-measurement state as

2 $${p}_{i}^{(k)}{\tilde{\rho }}_{{\rm{AB}}i}^{(k)}=({{\rm{\Pi }}}_{{\rm{A}}i}^{(k)}\otimes I){\rho }_{{\rm{A}}{\rm{B}}}({{\rm{\Pi }}}_{{\rm{A}}i}^{(k)}\otimes I)={p}_{i}^{(k)}{{i}^{(k)}}_{{\rm{A}}}{i}^{(k)}\otimes {\tilde{\rho }}_{{\rm{B}}i}^{(k)},$$

where *k* labels the reference basis, and the probability of obtaining a result *i* is ${p}_{i}^{(k)}={{\rm{Tr}}}_{{\rm{AB}}}({{\rm{\Pi }}}_{{\rm{A}}i}^{(k)}\otimes I){\rho }_{{\rm{A}}{\rm{B}}}({{\rm{\Pi }}}_{{\rm{A}}i}^{(k)}\otimes I)$. Then, thermal discord is defined as^34,36^

3 $${D}_{{\rm{th}}}^{(k)}({\rm{B}}|{\rm{A}})\,:\,=\sum _{i=1}^{d}\,{p}_{i}^{(k)}S({\tilde{\rho }}_{{\rm{B}}i}^{(k)})+S({\tilde{\rho }}_{{\rm{A}}}^{(k)})-S({\rho }_{{\rm{AB}}}).$$

with ${\tilde{\rho }}_{{\rm{Bi}}}^{(k)}={{\rm{Tr}}}_{{\rm{A}}}({{\rm{\Pi }}}_{{\rm{A}}i}^{(k)}\otimes I){\rho }_{{\rm{AB}}}({{\rm{\Pi }}}_{{\rm{A}}i}^{(k)}\otimes I)/{p}_{i}^{(k)}$ the post-measure state of subsystem B with probability ${p}_{i}^{(k)}$ and

4 $${\tilde{\rho }}_{{\rm{A}}}^{(k)}={{\rm{Tr}}}_{{\rm{B}}}\,[\sum _{i}({{\rm{\Pi }}}_{{\rm{A}}i}^{(k)}\otimes I){\rho }_{{\rm{AB}}}({{\rm{\Pi }}}_{{\rm{A}}i}^{(k)}\otimes I)]=\sum _{i}\,{p}_{i}^{(k)}{{i}^{(k)}}_{{\rm{A}}}{i}^{(k)},$$

the state of the subsystem A after the measurement without knowing any outcome. Thermal discord concerns about the entropic cost of performing a local projective measurement on the subsystem of a bipartite state and is relevant with the thermodynamics of correlated systems^36^. The definition of thermal discord is also measurement-dependent. We can generally make a measurement-independent definition by requiring a minimization over all projective measurements, but this would in principle complicate the calculation and make it difficult to obtain a closed expression. In the experiment, we would always choose specific measurements when the optimal measurement is not available. In the meantime to gain more information, one would implement more than one measurements with different bases. In such a situation, it would be very helpful if we know any relation among thermal discord with multiple measurements.

Moreover, conditional information on joint system AB is defined as *S*(A|B) := *S*(*ρ*_AB_) − *S*(*ρ*_B_)^37^. We denote the conditional entropy after a measurement ${ {\mathcal M} }_{{\rm{A}}}$ on the subsystem A as $S({ {\mathcal M} }_{{\rm{A}}}|{\rm{B}})=S({\tilde{\rho }}_{{\rm{AB}}})-S({\tilde{\rho }}_{{\rm{B}}})$. In this case, instead of multiple measurements on one system, we may have a complementary relation on post-measurement conditional information in a multipartite system.

### Complementary relation of quantum coherence

The corresponding post-measurement state for a measurement ${ {\mathcal M} }^{(k)}$ and a quantum state *ρ* can be written as,

5 $${\tilde{\rho }}^{(k)}=\sum _{i}{{\rm{\Pi }}}_{i}^{(k)}\rho {{\rm{\Pi }}}_{i}^{(k)}=\sum _{i}{p}_{i}^{(k)}{\tilde{\rho }}_{i}^{(k)},$$

which can be written in a more explicit form as, ${\tilde{\rho }}^{(k)}={\sum }_{i}{p}_{i}^{(k)}|{i}^{(k)}\rangle \langle {i}^{(k)}|$. The entropy of this post-measurement state takes the form,

6 $$S({\tilde{\rho }}^{(k)})=-\,{\rm{Tr}}{\tilde{\rho }}^{(k)}{\mathrm{log}}_{2}{\tilde{\rho }}^{(k)}=-\,\sum _{i}{p}_{i}^{(k)}{\mathrm{log}}_{{\rm{2}}}{p}_{i}^{(k)},$$

which describes how exactly we can measure the state *ρ* by the measurement operator ${ {\mathcal M} }^{(k)}$. For example, for a projective measurement in computational basis {|0〉,|1〉} on a qubit state, if we obtain the measurement results |0〉 and |1〉 with an equal probability 1/2, the entropy in Eq. (6) is 1. In this case, we do not know whether the state should be |0〉 or |1〉, since both the probabilities to obtain those two states are 1/2. The state corresponds to a completely mixed state or a maximally coherent state |+〉 or |−〉, where $|\pm \,\rangle =\,(|0\rangle +\mathrm{|1}\rangle )/\sqrt{2}$. If we obtain |0〉 or |1〉 with probability 1, the result of relation (6) is 0. We know exactly whether the state is |0〉 or |1〉. The principle of uncertainty in quantum mechanics states that we cannot achieve arbitrary measurement precision simultaneously for non-commuting observables, meaning that the summation of entropies for the post-measurement states for non-commuting observables should have a lower bound larger than 0 for an arbitrary quantum state. Generally, the entropic uncertainty relation with *N* measurements ${\{{ {\mathcal M} }^{(k)}\}}_{k=1}^{N}$ can be written as follows^51^

7 $$\sum _{k=1}^{N}S({\tilde{\rho }}^{(k)})\ge -\,{\mathrm{log}}_{2}b+(N-\mathrm{1)}S(\rho ),$$

where *b* ∈ (0, 1] is determined by measurement operators ${\{{ {\mathcal M} }^{(k)}\}}_{k=1}^{N}$,

8 $$b=[\mathop{{\rm{\max }}}\limits_{{i}_{N}}\sum _{{i}_{2},\ldots ,{i}_{N-1}}\mathop{{\rm{\max }}\,}\limits_{{i}_{1}}[{\rm{Tr}}({{\rm{\Pi }}}_{{i}_{1}}^{\mathrm{(1)}},{{\rm{\Pi }}}_{{i}_{2}}^{\mathrm{(2)}})]\times \prod _{k=2}^{N-1}{\rm{Tr}}({{\rm{\Pi }}}_{{i}_{k}}^{(k)},{{\rm{\Pi }}}_{{i}_{k+1}}^{(k+\mathrm{1)}})].$$

Here, ${\tilde{\rho }}^{(k)}$ is the post-measurement state for ${ {\mathcal M} }^{(k)}$, as we have mentioned, where ${\rm{Tr}}({{\rm{\Pi }}}_{{i}_{1}}^{\mathrm{(1)}},{{\rm{\Pi }}}_{{i}_{2}}^{\mathrm{(2)}})=|\langle {i}_{1}^{\mathrm{(1)}}|{i}_{2}^{\mathrm{(2)}}\rangle {|}^{2}$ corresponds to the square of the overlap between two different bases. We remark that quantity *b* depends only on the measurement set ${\{{ {\mathcal M} }^{(k)}\}}_{k\mathrm{=1}}^{N}$, and no order should be a priori assumed for ${\{{ {\mathcal M} }^{(k)}\}}_{k=1}^{N}$ in Eq. (8), so *b* is state independent. We adjust (7) to another form

9 $$\sum _{k=1}^{N}S({\tilde{\rho }}^{(k)})-S(\rho )\ge -\,{\mathrm{log}}_{2}b-S(\rho )$$

for ease of obtaining the complementary relation of quantum coherence. Since the relative entropy coherence measure ${C}_{{\rm{RE}}}^{(k)}(\rho )$ corresponding to the reference basis $\{{{\rm{\Pi }}}_{i}^{(k)}\}$ can be defined as the increase of the entropy *S*(*ρ*^(*k*)^) − *S*(*ρ*) of the system due to measurement ${ {\mathcal M} }^{(k)}$, we can equivalently obtain

10 $$\sum _{k=1}^{N}{C}_{{\rm{RE}}}^{(k)}(\rho )\ge -\,{\mathrm{log}}_{2}b-S(\rho ),$$

which provides a lower bound for the relative entropy of quantum coherence in addition to the upper bound given in ref.^22^. In particular, if the state is pure, *S*(*ρ*) = 0, the bound shown in the right-hand-side of the inequality (10) depends only on measurement bases, and can be larger than that of a mixed state. In this case, the bound can be a finite positive value if we choose different measurement bases, implying that the quantum coherence of a pure state can always be non-zero if we can choose an appropriate basis. We remark that the inequality (10) is simply a different form of the entropic uncertainty relation with multiple measurements^51^. However, the implication of this inequality is different as the inequality (10) is for total coherence with different bases. In particular, when there is no fixed basis in measuring coherence, a constraint in combining quantum coherence measured in different bases will be insightful. The complementary relation describes the properties of coherence from this point of view.

We know that the relative entropy of coherence ${C}_{{\rm{RE}}}^{(k)}(\rho )$ is non-negative, however, the right-hand-side of the inequality can be positive, zero or even negative. In particular, when *ρ* is a mixed state, *S*(*ρ*) can be relatively large, so the lower bound will probably be negative. This fact is reasonable because that coherence depends on the chosen basis, i.e., projective measurement ${\mathcal M}$. Let us consider an extreme case when *ρ* is a completely mixed state, *S*(*ρ*) = log_2_*d*, where *d* is the dimension of Hilbert space. The coherence is always zero regardless of the basis, so the total coherence in the left-hand-side of Eq. (10) is also zero, while right-hand-side is no larger than 0, −log_2_*b* − *S*(*ρ*) ≤ 0. Then, the inequality is always true for arbitrary sets of ${\{{ {\mathcal M} }^{(k)}\}}_{k=1}^{N}$. The equality can be satisfied for some specifically bases such as the mutually unbiased bases. This fact also indicates that we can expect a higher bound if the state has a larger purity resulting in a smaller entropy. We notice that this result (10) was also reported in ref.^53^ very recently.

For example, let us study a density matrix as follows,

11 $$\rho =p|\psi \rangle \langle \psi |+\frac{1-p}{2}{\bf{I}},$$

which is constituted by a maximally coherent state |*ψ*〉 and a completely mixed state **I**/2, where *p* ∈ [0, 1]. This maximally coherent state takes the form,

12 $$|\psi \rangle =\frac{1}{\sqrt{2}}\mathrm{(|0}\rangle +{e}^{i\varphi }\mathrm{|1}\rangle ),$$

where *ϕ* ∈ [0, 2*π*) is the phase parameter, and **I** is the identity operator in two-dimensional Hilbert space. We consider two measurement sets {|0〉, |1〉} and {|+〉, |−〉}, respectively. In this case, $b=\frac{1}{2}$, based on Eq. (8). The entropy of state *ρ* is,

13 $$S(\rho )=-\,\frac{1+p}{2}{\mathrm{log}}_{2}\frac{1+p}{2}-\frac{1-p}{2}{\mathrm{log}}_{2}\frac{1-p}{2}\mathrm{.}$$

The diagonal form of the density matrix can be written respectively in two bases, {|0〉, |1〉}, {|+〉, |−〉}, as follows,

14 $${\rho }_{diag\mathrm{.}}={\{\frac{1}{2},\frac{1}{2}\}}_{\mathrm{\{|0}\rangle \mathrm{,|1}\rangle \}},$$

15 $${\rho }_{diag\mathrm{.}}^{^{\prime} }={\{\frac{1+p\cos \varphi }{2},\frac{1-p\cos \varphi }{2}\}}_{\{|+\rangle ,|-\rangle \}}.$$

Thus, for the computational basis, the entropy of the diagonal density matrix is *S*(*ρ*_*diag*._) = 1. For the basis {|+〉, |−〉}, the entropy of the diagonal density matrix is,

16 $$S({\rho }_{diag\mathrm{.}}^{^{\prime} })=-\,\frac{1+p^{\prime} }{2}{\mathrm{log}}_{2}\frac{1+p^{\prime} }{2}-\frac{1-p^{\prime} }{2}{\mathrm{log}}_{2}\frac{1-p^{\prime} }{2},$$

where we denote *p*′ = *p* cos *ϕ* for convenience.

By using Eq. (7) and Eq. (10), the complementary relation for coherence implies the following inequality,

17 $$[S({\rho }_{diag\mathrm{.}})-S(\rho )]+[S({\rho }_{diag\mathrm{.}}^{^{\prime} })-S(\rho )]\ge -\,{\mathrm{log}}_{2}b-S(\rho \mathrm{).}$$

The bound in right-hand-side takes the value, 1 − *S*(*ρ*), meaning that the total coherence of state *ρ* based on two measurement bases should be larger than a positive value unless it becomes a completely mixed state which leads to *S*(*ρ*) = 1. On the other hand, for a pure state *ρ*, *S*(*ρ*) = 0, the bound equals to 1. So the total coherence should be always larger or equal to 1. Thus, we know that coherence of state *ρ* as a resource depends not only on basis, but also on the von Neumann entropy of the state.

Next, we show explicitly that this inequality is true. Substituting the results, *S*(*ρ*_*diag*._) = 1 and $b=\frac{1}{2}$, the inequality becomes,

18 $$S({\rho }_{diag\mathrm{.}}^{^{\prime} })-S(\rho )\ge 0.$$

By considering the results in Eqs (13) and (16), we know this inequality is correct based on the fact *p*′ ≤ *p*. When *ϕ* = 0 meaning *p* = *p*′, the inequality becomes an equality. This result can also be understood as the fact that the relative entropy of coherence for state *ρ* is nonnegative.

Although the quantum coherence measure is basis dependent, for multiple measurement bases, the summation of the coherence can be bounded from below by a quantity depending on *b* determined by the overlap between different bases and the entropy of the state. The parameter *b* itself is independent of the studied state. So there exists the complementary relation for coherence with multiple measurements.

### Complementary relations of thermal discord

For the quantum state *ρ*_AB_, in case the conditional entropy, also in name of conditional information, is negative, *S*(A|B) := *S*(*ρ*_AB_) − *S*(*ρ*_B_) < 0, we know that there is entanglement between A and B. In this case, suppose that subsystem B plays the role of quantum memory, the uncertainty extent of subsystem A for measurements will decrease^49^. In this physical setting, we perform multiple measurements on A, the multi-measurement uncertainty with the assistance of memory B can be derived as follows^51^

19 $$\sum _{k=1}^{N}S({ {\mathcal M} }_{{\rm{A}}}^{(k)}|{\rm{B}})\ge -\,{\mathrm{log}}_{2}b+(N-\mathrm{1)}S({\rm{A}}|{\rm{B}}).$$

where $S({ {\mathcal M} }_{{\rm{A}}}^{(k)}|{\rm{B}})=S({\tilde{\rho }}_{{\rm{AB}}}^{(k)})-S({\tilde{\rho }}_{{\rm{B}}}^{(k)})$ is the conditional information after the measurement ${ {\mathcal M} }_{{\rm{A}}}^{(k)}$ on A, recalling the notations and results in Eq. (2), and we have ${\tilde{\rho }}_{{\rm{AB}}}^{(k)}={\sum }_{i}{p}_{i}^{(k)}{\tilde{\rho }}_{{\rm{AB}}i}^{(k)}$. We can rewrite the first term of thermal discord in the definition (3) as

20 $$\begin{array}{rcl}\sum _{i\mathrm{=1}}^{d}{p}_{i}(k)S({\tilde{\rho }}_{{\rm{B}}i}^{(k)}) & = & S(\sum _{i=1}^{d}{p}_{i}^{(k)}{{\rm{\Pi }}}_{{\rm{A}}i}^{(k)}\otimes {\tilde{\rho }}_{{\rm{B}}i}^{(k)})-S({\tilde{\rho }}_{{\rm{A}}}^{(k)})\\  & = & S({\tilde{\rho }}_{{\rm{AB}}}^{(k)})-S({\tilde{\rho }}_{{\rm{A}}}^{(k)}\mathrm{).}\end{array}$$

We recall that ${\tilde{\rho }}_{{\rm{B}}i}^{(k)}$ is the reduced density operator of subsystem B corresponding to the result *i* after the measurement ${ {\mathcal M} }_{{\rm{A}}}^{(k)}$ on subsystem A. ${\tilde{\rho }}_{{\rm{A}}}^{(k)}$, ${\tilde{\rho }}_{{\rm{B}}}^{(k)}$ and ${\tilde{\rho }}_{{\rm{AB}}}^{(k)}$ stand for states of subsystems A, B and joint system AB, respectively, after the measurement ${ {\mathcal M} }_{{\rm{A}}}^{(k)}$. Therefore, thermal discord can be expressed as

21 $${D}_{{\rm{th}}}^{(k)}({\rm{B}}|{\rm{A}})=S({\tilde{\rho }}_{{\rm{AB}}}^{(k)})-S({\tilde{\rho }}_{{\rm{A}}}^{(k)})+S({\tilde{\rho }}_{{\rm{A}}}^{(k)})-S({\rho }_{{\rm{AB}}})=[S({\tilde{\rho }}_{{\rm{AB}}}^{(k)})-S({\rho }_{{\rm{B}}})]-[S({\rho }_{{\rm{AB}}})-S({\rho }_{{\rm{B}}})]=S({ {\mathcal M} }_{{\rm{A}}}^{(k)}|{\rm{B}})-S({\rm{A}}|{\rm{B}}),$$

where we have used the fact that the projective measurements are performed on subsystem A leading to the equality, ${{\rm{Tr}}}_{{\rm{A}}}{\tilde{\rho }}_{{\rm{AB}}}^{(k)}={{\rm{Tr}}}_{{\rm{A}}}{\rho }_{{\rm{AB}}}={\rho }_{{\rm{B}}}$. It is now straightforward to rewrite Eq. (19) as

22 $$\sum _{k\mathrm{=1}}^{N}{D}_{{\rm{th}}}^{(k)}({\rm{B}}|{\rm{A}})\ge -\,{\mathrm{log}}_{2}b-S({\rm{A}}|{\rm{B}}),$$

which serves as a complementary relation for thermal discord with respect to different measurement bases. This lower bound contains a term of conditional information of the pre-measurement state, and thus is also state dependent. As we have mentioned the negativity of conditional information signals entanglement, for a more entangled state, we can expect higher thermal discord. We remark that the inequality (22), resulting from the entropic uncertainty relation with the assistance of a quantum memory, implies that thermal discord as a resource for different bases is larger than a bound depending on both measurement bases and the bipartite state. It will be interesting if applications of this inequality can be found in statistical mechanics^34^.

One would notice that the relation (22) would reduce to (10) when the dimension of the Hilbert space of B is reduced to 1. This indicates a close relation between the relative entropy coherence and thermal discord. In this case, thermal discord would reduce to a relative entropy coherence measure, and conditional information *S*(A|B) would equal to *S*(*ρ*_A_) since *ρ*_AB_ = *ρ*_A_ and *ρ*_B_ = 1.

### Complementary relation of post-measurement conditional information

Next, we consider a multipartite system, including *N* + 2 parties AB_0_ ··· B_*N*_. We would consider *N* + 1 measurements ${ {\mathcal M} }_{{\rm{A}}}^{(k)}$ on the subsystem A. It means the number of multiple measurements performed on A is equal to the number of subsystems *B*_0_*B*_1_…*B*_*N*_. By introducing an ancillary subsystem C, we can always purify ${\rho }_{{{\rm{AB}}}_{0}{{\rm{B}}}_{k}}$ to ${\rho }_{{{\rm{AB}}}_{0}{{\rm{B}}}_{k}{\rm{C}}}=(|{\rm{\Psi }}\rangle {\langle {\rm{\Psi }}|)}_{{{\rm{AB}}}_{0}{{\rm{B}}}_{k}{\rm{C}}}$, satisfying ${\rho }_{{{\rm{AB}}}_{0}{{\rm{B}}}_{k}}={{\rm{Tr}}}_{{\rm{C}}}(|{\rm{\Psi }}\rangle {\langle {\rm{\Psi }}|)}_{{{\rm{AB}}}_{0}{{\rm{B}}}_{k}{\rm{C}}}$. The projective measurement is still performed on subsystem A. Specifically, for the *k*-th measurement ${ {\mathcal M} }_{{\rm{A}}}^{(k)}$, we can use the corresponding purified state to express the measurement as,

23 $$({{\rm{\Pi }}}_{{\rm{A}}i}^{(k)}\otimes {I}_{{{\rm{B}}}_{0}{{\rm{B}}}_{k}{\rm{C}}})|{\rm{\Psi }}{\rangle }_{{{\rm{AB}}}_{0}{{\rm{B}}}_{k}{\rm{C}}}=\sqrt{{p}_{i}^{(k)}}|{i}^{(k)}{\rangle }_{{\rm{A}}}|{\tilde{{\rm{\Psi }}}}_{i}{\rangle }_{{{\rm{B}}}_{0}{{\rm{B}}}_{k}{\rm{C}}},$$

which shows that the post-measurement state of B_0_B_*k*_C corresponding to result *i* of measurement ${ {\mathcal M} }_{{\rm{A}}}^{(k)}$ on subsystem A is a pure state with the form $|{\tilde{{\rm{\Psi }}}}_{i}{\rangle }_{{{\rm{B}}}_{0}{{\rm{B}}}_{k}{\rm{C}}}$. It is known that for the pure state $|{\tilde{{\rm{\Psi }}}}_{i}{\rangle }_{{{\rm{B}}}_{0}{{\rm{B}}}_{k}{\rm{C}}}$, based on Schmidt decomposition^37^, the von Neumann entropy of the two reduced density matrices by partition B_0_B_*k*_:C are the same,

24 $$S({\tilde{\rho }}_{{{\rm{B}}}_{0i}})=S({\tilde{\rho }}_{{{\rm{B}}}_{k}{{\rm{C}}}_{i}})\mathrm{.}$$

In addition, still based on Schmidt decomposition, the two density matrices can be transferred to each other by a unitary transformation. Similarly, let us consider the pure state $|{\rm{\Psi }}{\rangle }_{{{\rm{AB}}}_{0}{{\rm{B}}}_{k}{\rm{C}}}$, we also know,

25 $$S({\rho }_{{{\rm{AB}}}_{0}})=S({\rho }_{{{\rm{B}}}_{k}{\rm{C}}}).$$

By taking summation over *i* for Eq. (23), we have the relation below,

26 $$\begin{array}{c}\sum _{i}({{\rm{\Pi }}}_{{\rm{A}}i}^{(k)}\otimes {I}_{{{\rm{B}}}_{0}{{\rm{B}}}_{k}{\rm{C}}})|{\rm{\Psi }}{\rangle }_{{{\rm{AB}}}_{0}{{\rm{B}}}_{k}{\rm{C}}}\\ =\sum _{i}\sqrt{{p}_{i}^{(k)}}|{i}^{(k)}{\rangle }_{{\rm{A}}}|{\tilde{{\rm{\Psi }}}}_{i}{\rangle }_{{{\rm{B}}}_{0}{{\rm{B}}}_{k}{\rm{C}}}.\end{array}$$

Starting from this state, with the help of the obtained results in Eqs (24 and 25), we can find,

27 $$\begin{array}{c}S({ {\mathcal M} }_{{\rm{A}}}^{(k)}|{{\rm{B}}}_{0})-S({\rm{A}}|{{\rm{B}}}_{0})=S(\sum _{i=1}^{d}{p}_{i}{{\rm{\Pi }}}_{{\rm{A}}i}^{(k)}\otimes {\tilde{\rho }}_{{{\rm{B}}}_{0}i}^{(k)})-S({\rho }_{{{\rm{AB}}}_{0}})\\ =S(\sum _{i=1}^{d}{p}_{i}{{\rm{\Pi }}}_{{\rm{A}}i}^{(k)}\otimes {\tilde{\rho }}_{{{\rm{B}}}_{k}{{\rm{C}}}_{i}}^{(k)})-S({\rho }_{{{\rm{B}}}_{k}{\rm{C}}})\le S(\sum _{i\mathrm{=1}}^{d}{p}_{i}{{\rm{\Pi }}}_{{\rm{A}}i}^{(k)}\otimes {\tilde{\rho }}_{{{\rm{B}}}_{k}i}^{(k)})-S({\rho }_{{{\rm{B}}}_{k}})\\ =S({ {\mathcal M} }_{{\rm{A}}}^{(k)}|{{\rm{B}}}_{k}),\end{array}$$

where the inequality is due to the strong subadditivity of the von Neumann entropy^37^, $$S({\rho }_{{{\rm{AB}}}_{k}{\rm{C}}})+S({\rho }_{{{\rm{B}}}_{k}})\le $$$$S({\rho }_{{{\rm{AB}}}_{k}})+S({\rho }_{{{\rm{B}}}_{k}{\rm{C}}})$$ leading to $$S({\rho }_{{{\rm{AB}}}_{k}{\rm{C}}})-S({\rho }_{{{\rm{B}}}_{k}{\rm{C}}})\le S({\rho }_{{{\rm{AB}}}_{k}})-S({\rho }_{{{\rm{B}}}_{k}})$$. Now by taking the summation for *k* = 1, 2, …, *N* on both sides of the inequality (27), we find,

28 $$\sum _{k=1}^{N}S({ {\mathcal M} }_{{\rm{A}}}^{(k)}|{{\rm{B}}}_{0})-NS({\rm{A}}|{{\rm{B}}}_{0})\le \sum _{k=1}^{N}S({ {\mathcal M} }_{{\rm{A}}}^{(k)}|{{\rm{B}}}_{k}\mathrm{).}$$

On the other hand, with the help of Eq. (19) where we replace *B* by *B*_0_, the above inequality (28) leads to,

29 $$S({\rm{A}}|{{\rm{B}}}_{0})+\sum _{k\mathrm{=1}}^{N}S({ {\mathcal M} }_{{\rm{A}}}^{(k)}|{{\rm{B}}}_{k})\ge -\,{\mathrm{log}}_{2}b.$$

Also we know that projective measurements can increase the entropy, and as a consequence, we have,

30 $$S({ {\mathcal M} }_{{\rm{A}}}^{(k)}|{{\rm{B}}}_{0})-S({\rm{A}}|{{\rm{B}}}_{0})=S({\tilde{\rho }}_{{{\rm{AB}}}_{0}}^{(k)})-S({\rho }_{{{\rm{AB}}}_{0}})\ge 0.$$

Thus, substituting *S*(A|B_0_) by $S({ {\mathcal M} }_{{\rm{A}}}^{(k)}|{{\rm{B}}}_{0})$, we can further obtain the complementary relation of post-measurements conditional information $S({ {\mathcal M} }_{{\rm{A}}}^{(k)}|{{\rm{B}}}_{k})$ as

31 $$\sum _{k=0}^{N}S({ {\mathcal M} }_{{\rm{A}}}^{(k)}|{{\rm{B}}}_{k})\ge -{\mathrm{log}}_{2}b,$$

which is similar to Eq. (10) of the relative entropy coherence measure and Eq. (22) of thermal discord. One merit of this bound is that it is state independent. Also it describes a constraint on bipartite quantum correlations in a multipartite system (usually with more than two subsystems), which is quite significant. One can also notice that when *N* = 1, this inequality can reduce to the uncertainty relation in ref.^50^ with two measurements for a tripartite state. Our results in this section may stimulate study of quantum correlations concerning multipartite systems.

## Discussion

By utilizing the uncertainty principle formulated in terms of entropies, we explicitly give a group of complementary relations for quantum coherence, thermal discord and conditional information. Specifically, the entropic uncertainty relation without a quantum memory would give us a lower bound (10) on quantum coherence in the system of a single particle with multiple measurements. It shows that the summation of the coherence measure of a quantum state with respect to different measurement bases should have a lower bound. Also the purer the state which corresponds to a lower entropy, the higher this bound would be. These entropic uncertainty relations concerning additional memories provide a lower bound in Eq. (22) on thermal discord with respect to different projective measurements on a subsystem of a bipartite joint system. This lower bound is for the summation of thermal discord with respect to multiple measurements on the chosen subsystem. It indicates that the more entanglement of the state which corresponds to higher minus conditional information, −*S*(A|B), the higher this lower bound would be. The latter group of complementary relations about thermal discord can be reduced to that of quantum coherence by discarding the auxiliary memory through setting its dimension to 1. In addition, we also derive a state-independent lower bound for the sum of post-measurement conditional information between a specific subsystem and the other subsystems. These three complementary relations indicate that there are important constraints for quantum quantities with multiple measurements. The results also imply that there exits a delicate relation between uncertainty principle with quantum coherence and quantum correlations in quantum mechanics.
